# Supplementary figures and images for: SWATH-MS based proteomic profiling of pancreatic ductal adenocarcinoma tumours reveals the interplay between the extracellular matrix and related intracellular pathways
Source: PLoS One. 2020 Oct 13;15(10):e0240453. doi: 10.1371/journal.pone.0240453 (PMC7553299; doi:10.1371/journal.pone.0240453)

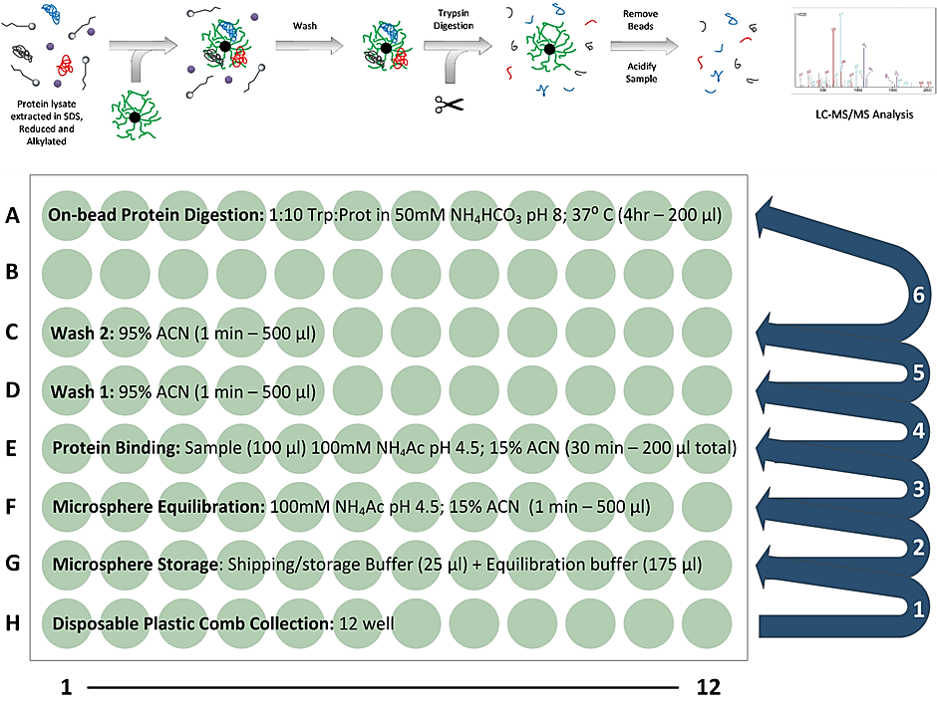

Supplement: S1 Fig — Top: proteins are captured on magnetic hydrophilic affinity microparticles, followed by a high-organic wash to remove contaminants and on-bead digestion by addition of sequencing grade trypsin. Bottom: Plate set-up for automated sample processing in a Thermo Fisher Scientific KingFisher™ Duo Magnetic handling station. (TIF) [file pone.0240453.s001.tif]

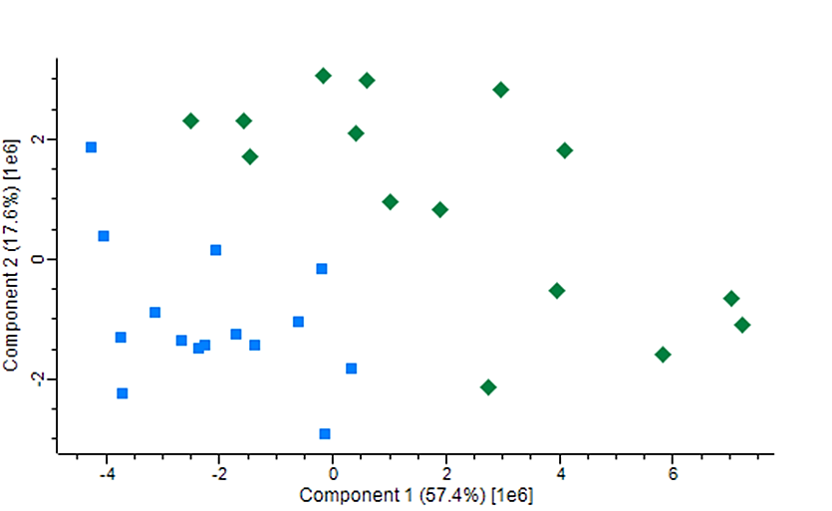

Supplement: S2 Fig — The PCA plot was generated using peptide abundance data of all peptides analysed per sample. (TIF) [file pone.0240453.s002.tif]

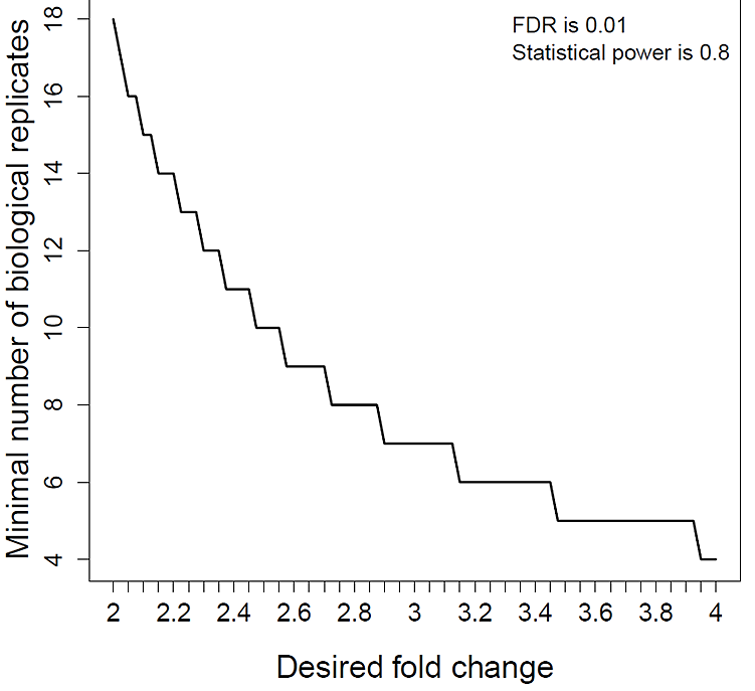

Supplement: S3 Fig — A 2.1 fold change can be distinguished in the current study (biological replicates = 15) with statistical power set to 0.8 and false discovery rate (FDR) set to 0.01. (TIF) [file pone.0240453.s003.tif]

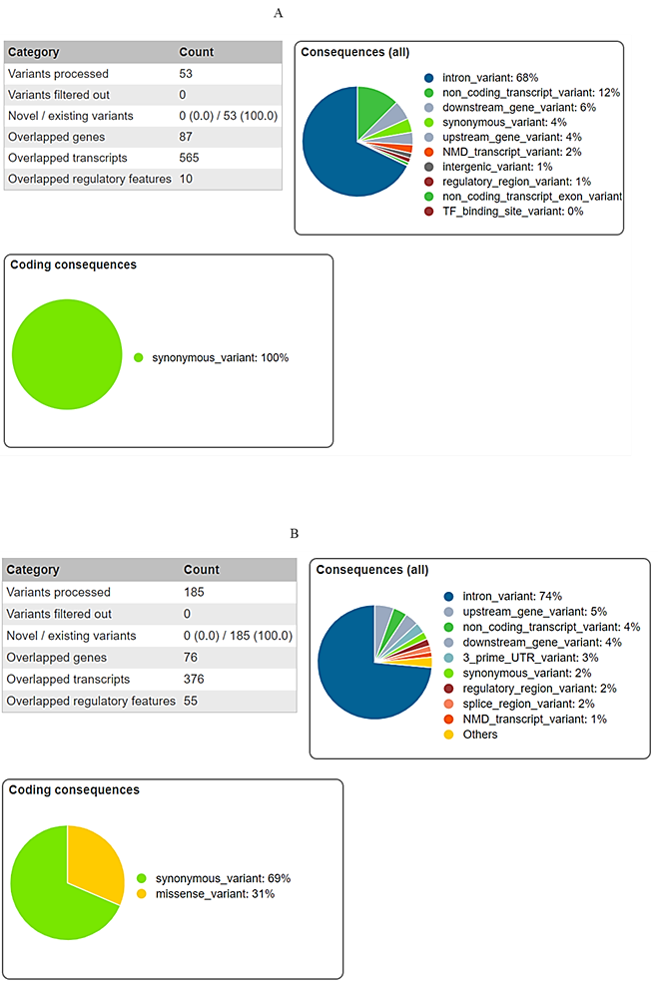

Supplement: S4 Fig — (TIF) [file pone.0240453.s004.tif]
