# Supplementary material for: SWATH-MS based proteomic profiling of pancreatic ductal adenocarcinoma tumours reveals the interplay between the extracellular matrix and related intracellular pathways
Source: PLoS One. 2020 Oct 13;15(10):e0240453. doi: 10.1371/journal.pone.0240453 (PMC7553299; doi:10.1371/journal.pone.0240453)
Supplement: S1 Table — (DOCX) [file pone.0240453.s005.docx]

**S1 Table**. **Demographic characteristics of patients recruited for the study.**

| **Patient** | **Gender** | **Age** | **Smoking** | **Alcohol use** | **Staging** | **TNM** |
| --- | --- | --- | --- | --- | --- | --- |
| 1 | Female | 95 | No | No | IIA | T2N1M0 |
| 2 | Male | 69 | Yes | Yes | IIA | T2N1M0 |
| 3 | Male | 68 | No | Yes | IIA | T2N1M0 |
| 4 | Male | 56 | Yes | Yes | IA | T1N0N0 |
| 5 | Female | 53 | No | No | IIB | T3N1M0 |
| 6 | Male | 54 | Yes | No | IIA | T2N1M0 |
| 7 | Male | 54 | No | Yes | IIA | T2N1M0 |
| 8 | Male | 56 | No | No | IIB | T3N1M0 |
| 9 | Female | 54 | No | Yes | IA | T1N0N0 |
| 10 | Male | 58 | Yes | Yes | IIA | T2N1M0 |
| 11 | Male | 74 | No | No | IIB | T3N1M0 |
| 12 | Female | 57 | Yes | Yes | IIA | T2N1M0 |
| 13 | Female | 61 | No | Yes | IIB | T3N1M0 |
| 14 | Female | 68 | No | Yes | IIB | T3N1M0 |
| 15 | Female | 65 | No | No | IIB | T3N1M0 |

TNM: TNM Classification of Malignant Tumors
