# Supplementary material for: SWATH-MS based proteomic profiling of pancreatic ductal adenocarcinoma tumours reveals the interplay between the extracellular matrix and related intracellular pathways
Source: PLoS One. 2020 Oct 13;15(10):e0240453. doi: 10.1371/journal.pone.0240453 (PMC7553299; doi:10.1371/journal.pone.0240453)
Supplement: S2 Table — Positive fold change describes proteins upregulated in the tumour group and negative fold change describes proteins downregulated in tumour compared to the normal adjacent group. (DOCX) [file pone.0240453.s006.docx]

**S2 Table. Differentially expressed proteins (fold change ≥2.1) measured in group comparison. Positive fold change describes proteins upregulated in the tumour group and negative fold change describes proteins downregulated in tumour compared to the normal adjacent group.**

| **Uni­prot ID** | **Uniprot Name** | **Fold change** | **Adjusted p-value** | **Location** | **Cancer (prognostic) biomarker status** | **Protein atlas reference link** |
| --- | --- | --- | --- | --- | --- | --- |
| Q86TC9 | MYPN_HUMAN | 6,90 | 4,71E-03 | Intracellular | Not prognostic | <https://www.proteinatlas.org/ENSG00000138347-MYPN> |
| P21980 | TGM2_HUMAN | 6,04 | 2,63E-03 | Intracellular | Unfavourable prognostic marker for renal and pancreatic cancers | <https://www.proteinatlas.org/ENSG00000198959-TGM2> |
| P62736 | ACTA_HUMAN | 5,96 | 9,81E-03 | Intracellular | Unfavourable prognostic marker for renal cancer | <https://www.proteinatlas.org/ENSG00000107796-ACTA2> |
| Q8WX93 | PALLD_HUMAN | 5,65 | 7,93E-03 | Intracellular | Unfavourable prognostic marker for urothelial cancer | <https://www.proteinatlas.org/ENSG00000129116-PALLD> |
| P50452 | SPB8_HUMAN | 5,47 | 4,71E-03 | Intracellular | Unfavourable prognostic marker for renal cancer | <https://www.proteinatlas.org/ENSG00000166401-SERPINB8> |
| O14498 | ISLR_HUMAN | 5,29 | 9,15E-03 | Secreted | Unfavourable prognostic marker for renal cancer | <https://www.proteinatlas.org/ENSG00000129009-ISLR> |
| P21810 | PGS1_HUMAN | 4,81 | 7,05E-03 | Intracellular | Unfavourable prognostic marker for renal cancer | <https://www.proteinatlas.org/ENSG00000182492-BGN> |
| A0AVI2 | FR1L5_HUMAN | 4,69 | 9,02E-03 | Intracellular | Not prognostic | <https://www.proteinatlas.org/ENSG00000249715-FER1L5> |
| Q9UHB6 | LIMA1_HUMAN | 4,52 | 7,80E-03 | Intracellular | Prognostic marker for head and neck cancers (unfavourable) and for renal cancer (favourable) | <https://www.proteinatlas.org/ENSG00000050405-LIMA1> |
| A0FGR8 | ESYT2_HUMAN | 4,00 | 3,16E-03 | Membrane | Unfavourable prognostic marker for urothelial and pancreatic cancers | <https://www.proteinatlas.org/ENSG00000117868-ESYT2> |
| Q9Y6C2 | EMIL1_HUMAN | 3,88 | 9,16E-03 | Intracellular | Unfavourable prognostic marker for renal cancer | <https://www.proteinatlas.org/ENSG00000138080-EMILIN1> |
| P63261 | ACTG_HUMAN | 3,72 | 1,66E-03 | Intracellular | Favourable prognostic marker for colorectal cancer | <https://www.proteinatlas.org/ENSG00000184009-ACTG1> |
| Q9NRN5 | OLFL3_HUMAN | 3,47 | 2,16E-03 | Intracellular/Secreted | Unfavourable prognostic marker for renal cancer | <https://www.proteinatlas.org/ENSG00000116774-OLFML3> |
| P48059 | LIMS1_HUMAN | 3,33 | 6,84E-03 | Intracellular | Unfavourable prognostic marker for renal cancer | <https://www.proteinatlas.org/ENSG00000169756-LIMS1> |
| P61769 | B2MG_HUMAN | 3,22 | 8,22E-03 | Intracellular | Not prognostic | <https://www.proteinatlas.org/ENSG00000166710-B2M> |
| P09936 | UCHL1_HUMAN | 3,15 | 6,58E-03 | Intracellular | Unfavourable prognostic marker for urothelial and endometrial cancers | <https://www.proteinatlas.org/ENSG00000154277-UCHL1> |
| P23634 | AT2B4_HUMAN | 3,07 | 9,73E-03 | Intracellular | Unfavourable prognostic marker for renal cancer | <https://www.proteinatlas.org/ENSG00000058668-ATP2B4> |
| Q16658 | FSCN1_HUMAN | 3,05 | 3,61E-03 | Intracellular | Unfavourable prognostic marker for renal, lung, head and neck cancers | <https://www.proteinatlas.org/ENSG00000075618-FSCN1> |
| P07858 | CATB_HUMAN | 3,03 | 7,37E-03 | Intracellular | Prognostic marker for thyroid (favourable) and urothelial (unfavourable) cancers | <https://www.proteinatlas.org/ENSG00000164733-CTSB> |
| P13796 | PLSL_HUMAN | 2,99 | 5,14E-03 | Intracellular | Not prognostic | <https://www.proteinatlas.org/ENSG00000136167-LCP1> |
| Q13308 | PTK7_HUMAN | 2,89 | 8,64E-03 | Intracellular/Secreted/Membrane | Not prognostic | <https://www.proteinatlas.org/ENSG00000112655-PTK7> |
| Q9H0R5 | GBP3_HUMAN | 2,86 | 4,44E-03 | Intracellular | Not prognostic | <https://www.proteinatlas.org/ENSG00000117226-GBP3> |
| Q16555 | DPYL2_HUMAN | 2,85 | 8,79E-04 | Intracellular/Membrane | Unfavourable prognostic marker for urothelial cancer | <https://www.proteinatlas.org/ENSG00000092964-DPYSL2> |
| P13929 | ENOB_HUMAN | 2,83 | 7,11E-04 | Intracellular | Unfavourable prognostic marker for colorectal cancer | <https://www.proteinatlas.org/ENSG00000108515-ENO3> |
| Q14141 | SEPT6_HUMAN | 2,83 | 2,27E-03 | Intracellular | Not prognostic | <https://www.proteinatlas.org/ENSG00000125354-SEPT6> |
| Q3ZCM7 | TBB8_HUMAN | 2,56 | 3,83E-03 | Intracellular | Not prognostic | <https://www.proteinatlas.org/ENSG00000261456-TUBB8> |
| Q14764 | MVP_HUMAN | 2,53 | 2,47E-03 | Intracellular | Favourable prognostic marker for renal and breast cancers | <https://www.proteinatlas.org/ENSG00000013364-MVP> |
| P17655 | CAN2_HUMAN | 2,52 | 3,21E-03 | Intracellular | Prognostic marker for renal (favourable), urothelial and pancreatic (unfavourable) cancers | <https://www.proteinatlas.org/ENSG00000162909-CAPN2> |
| P12814 | ACTN1_HUMAN | 2,49 | 8,79E-04 | Intracellular | Unfavourable prognostic marker for renal, lung, urothelial, head and neck cancers | <https://www.proteinatlas.org/ENSG00000072110-ACTN1> |
| P62873 | GBB1_HUMAN | 2,49 | 3,41E-04 | Intracellular/Secreted | Unfavourable prognostic marker for liver cancer | <https://www.proteinatlas.org/ENSG00000078369-GNB1> |
| O94973 | AP2A2_HUMAN | 2,48 | 3,71E-04 | Intracellular | Prognostic marker for liver (favourable) and pancreatic (unfavourable) cancers | <https://www.proteinatlas.org/ENSG00000183020-AP2A2> |
| Q9UPN3 | MACF1_HUMAN | 2,43 | 1,66E-03 | Intracellular | Favourable prognostic marker for renal cancer | <https://www.proteinatlas.org/ENSG00000127603-MACF1> |
| Q9NZU5 | LMCD1_HUMAN | 2,43 | 7,16E-03 | Intracellular | Unfavourable prognostic marker for renal cancer | <https://www.proteinatlas.org/ENSG00000071282-LMCD1> |
| Q9BUF5 | TBB6_HUMAN | 2,43 | 1,53E-03 | Intracellular | Unfavourable prognostic marker for renal and urothelial cancers | <https://www.proteinatlas.org/ENSG00000176014-TUBB6> |
| Q15149 | PLEC_HUMAN | 2,42 | 8,49E-04 | Intracellular | Unfavourable prognostic marker for lung, colorectal and renal cancers | <https://www.proteinatlas.org/ENSG00000178209-PLEC> |
| Q13885 | TBB2A_HUMAN | 2,38 | 1,35E-03 | Intracellular | Prognostic marker for renal (favourable), urothelial (unfavourable) cancers | <https://www.proteinatlas.org/ENSG00000137267-TUBB2A> |
| Q01518 | CAP1_HUMAN | 2,34 | 6,56E-03 | Intracellular | Unfavourable prognostic marker for liver cancer | <https://www.proteinatlas.org/ENSG00000131236-CAP1> |
| P35579 | MYH9_HUMAN | 2,31 | 9,71E-03 | Intracellular | Not prognostic | <https://www.proteinatlas.org/ENSG00000100345-MYH9> |
| P53634 | CATC_HUMAN | 2,29 | 8,65E-03 | Intracellular | Prognostic marker for renal (favourable) and liver (unfavourable) cancers | <https://www.proteinatlas.org/ENSG00000109861-CTSC> |
| P62879 | GBB2_HUMAN | 2,26 | 8,79E-04 | Intracellular | Not prognostic | <https://www.proteinatlas.org/ENSG00000172354-GNB2> |
| O75083 | WDR1_HUMAN | 2,25 | 2,59E-04 | Intracellular | Favourable prognostic marker for renal cancer | <https://www.proteinatlas.org/ENSG00000071127-WDR1> |
| P13797 | PLST_HUMAN | 2,22 | 3,29E-03 | Intracellular | Unfavourable prognostic marker for renal, thyroid, pancreatic, urothelial, head and neck cancers | <https://www.proteinatlas.org/ENSG00000102024-PLS3> |
| A1X283 | SPD2B_HUMAN | 2,21 | 2,64E-03 | Intracellular | Unfavourable prognostic marker for liver, renal, urothelial, cancers | <https://www.proteinatlas.org/ENSG00000174705-SH3PXD2B> |
| Q13418 | ILK_HUMAN | 2,20 | 3,63E-03 | Intracellular | Favourable prognostic marker for renal cancer | <https://www.proteinatlas.org/ENSG00000166333-ILK> |
| P52943 | CRIP2_HUMAN | 2,20 | 9,71E-03 | Intracellular | Not prognostic | <https://www.proteinatlas.org/ENSG00000182809-CRIP2> |
| Q96RF0 | SNX18_HUMAN | 2,18 | 7,37E-03 | Intracellular | Favourable prognostic marker for renal cancer | <https://www.proteinatlas.org/ENSG00000178996-SNX18> |
| Q14651 | PLSI_HUMAN | 2,18 | 1,06E-03 | Intracellular | Prognostic marker for renal (favourable) and pancreatic (unfavourable) cancers | <https://www.proteinatlas.org/ENSG00000120756-PLS1> |
| Q9Y4F1 | FARP1_HUMAN | 2,16 | 5,55E-04 | Intracellular | Favourable prognostic marker for renal cancer | <https://www.proteinatlas.org/ENSG00000152767-FARP1> |
| P07437 | TBB5_HUMAN | 2,13 | 5,44E-03 | Intracellular | Unfavourable prognostic marker for liver and renal cancers | <https://www.proteinatlas.org/ENSG00000196230-TUBB> |
| Q00013 | EM55_HUMAN | -2,15 | 5,92E-03 | Intracellular/Membrane | Favourable prognostic marker for renal cancer | <https://www.proteinatlas.org/ENSG00000130830-MPP1> |
| P13716 | HEM2_HUMAN | -2,15 | 8,59E-04 | Intracellular/Secreted | Prognostic marker for liver, renal, endometrial (favourable) and colorectal (unfavourable) cancers | <https://www.proteinatlas.org/ENSG00000148218-ALAD> |
| Q8N183 | MIMIT_HUMAN | -2,19 | 1,66E-03 | Intracellular | Unfavourable prognostic marker for liver and renal cancers | <https://www.proteinatlas.org/ENSG00000164182-NDUFAF2> |
| Q8IZ83 | A16A1_HUMAN | -2,28 | 3,41E-04 | Intracellular | Not prognostic | <https://www.proteinatlas.org/ENSG00000161618-ALDH16A1> |
| O94875 | SRBS2_HUMAN | -2,32 | 5,14E-03 | Intracellular | Favourable prognostic marker for endometrial, liver and renal cancer | <https://www.proteinatlas.org/ENSG00000154556-SORBS2> |
| P48506 | GSH1_HUMAN | -2,38 | 1,70E-04 | Intracellular | Prognostic marker for renal (favourable) and thyroid (unfavourable) cancers | <https://www.proteinatlas.org/ENSG00000001084-GCLC> |
| P61960 | UFM1_HUMAN | -2,42 | 9,71E-03 | Intracellular | Prognostic marker for endometrial (favourable) and head and neck (unfavourable) cancers | <https://www.proteinatlas.org/ENSG00000120686-UFM1> |
| P30740 | ILEU_HUMAN | -2,42 | 9,71E-03 | Intracellular | Not prognostic | <https://www.proteinatlas.org/ENSG00000021355-SERPINB1> |
| P17213 | BPI_HUMAN | -2,47 | 8,05E-03 | Intracellular/Secreted | Not prognostic | <https://www.proteinatlas.org/ENSG00000101425-BPI> |
| P16219 | ACADS_HUMAN | -2,52 | 7,80E-03 | Intracellular | Favourable prognostic marker for renal, endometrial, liver cancers | <https://www.proteinatlas.org/ENSG00000122971-ACADS> |
| P08397 | HEM3_HUMAN | -2,54 | 5,14E-03 | Intracellular | Not prognostic | <https://www.proteinatlas.org/ENSG00000256269-HMBS> |
| P54687 | BCAT1_HUMAN | -2,87 | 5,81E-03 | Intracellular | Unfavourable prognostic marker for renal, stomach, urothelial, head and neck cancers | <https://www.proteinatlas.org/ENSG00000060982-BCAT1> |
| P35580 | MYH10_HUMAN | -2,88 | 5,55E-04 | Intracellular | Prognostic marker for renal (favourable) and urothelial (unfavourable) cancers | <https://www.proteinatlas.org/ENSG00000133026-MYH10> |
| Q7Z2W4 | ZCCHV_HUMAN | -2,90 | 5,40E-03 | Intracellular | Prognostic marker for stomach (favourable) and liver (unfavourable) cancers | <https://www.proteinatlas.org/ENSG00000105939-ZC3HAV1> |
| Q14766 | LTBP1_HUMAN | -2,98 | 8,05E-03 | Intracellular/Secreted | Unfavourable prognostic marker for renal and urothelial cancers | <https://www.proteinatlas.org/ENSG00000049323-LTBP1> |
| P10412 | H14_HUMAN | -3,02 | 8,79E-04 | Intracellular | Not prognostic | <https://www.proteinatlas.org/ENSG00000168298-HIST1H1E> |
| P30043 | BLVRB_HUMAN | -3,23 | 1,40E-04 | Intracellular | Unfavourable prognostic marker for pancreatic cancer | <https://www.proteinatlas.org/ENSG00000090013-BLVRB> |
| P04040 | CATA_HUMAN | -3,28 | 4,46E-04 | Intracellular | Favourable prognostic marker for renal and liver cancer | <https://www.proteinatlas.org/ENSG00000121691-CAT> |
| P62891 | RL39_HUMAN | -3,35 | 8,05E-03 | Intracellular/Secreted | Unfavourable prognostic marker for renal cancer | <https://www.proteinatlas.org/ENSG00000198918-RPL39> |
| P68871 | HBB_HUMAN | -3,67 | 1,92E-04 | Intracellular | Not prognostic | <https://www.proteinatlas.org/ENSG00000244734-HBB> |
| P32119 | PRDX2_HUMAN | -3,81 | 2,59E-04 | Intracellular | Favourable prognostic marker for renal cancer | <https://www.proteinatlas.org/ENSG00000167815-PRDX2> |
| Q08495 | DEMA_HUMAN | -3,90 | 2,56E-03 | Intracellular | Prognostic marker for renal (favourable) and glioma(unfavourable) cancers | <https://www.proteinatlas.org/ENSG00000158856-DMTN> |
| P11166 | GTR1_HUMAN | -3,94 | 2,08E-03 | Intracellular/Membrane | Unfavourable prognostic marker for renal, liver, lung, pancreatic and urothelial cancers | <https://www.proteinatlas.org/ENSG00000117394-SLC2A1> |
| P69905 | HBA_HUMAN | -4,01 | 2,98E-04 | Intracellular | Not prognostic | <https://www.proteinatlas.org/ENSG00000206172-HBA1> |
| P07738 | PMGE_HUMAN | -4,12 | 8,59E-04 | Intracellular | Favourable prognostic marker for renal and cervical cancers | <https://www.proteinatlas.org/ENSG00000172331-BPGM> |
| P02042 | HBD_HUMAN | -4,27 | 1,70E-04 | Intracellular/Membrane | Not prognostic | <https://www.proteinatlas.org/ENSG00000223609-HBD> |
| P00918 | CAH2_HUMAN | -4,52 | 4,46E-04 | Intracellular/Membrane | Favourable prognostic marker for renal cancer | <https://www.proteinatlas.org/ENSG00000104267-CA2> |
| P49913 | CAMP_HUMAN | -4,89 | 6,40E-03 | Secreted | Favourable prognostic marker for cervical cancer | <https://www.proteinatlas.org/ENSG00000164047-CAMP> |
| Q8IY47 | KBTB2_HUMAN | -5,42 | 5,55E-04 | Intracellular | Unfavourable prognostic marker for liver and cervical cancers | <https://www.proteinatlas.org/ENSG00000170852-KBTBD2> |
| Q96C19 | EFHD2_HUMAN | -5,49 | 6,56E-03 | Intracellular | Prognostic marker for urothelial, thyroid (favourable), lung and renal (unfavourable) cancers | <https://www.proteinatlas.org/ENSG00000142634-EFHD2> |
| P08514 | ITA2B_HUMAN | -6,43 | 3,09E-03 | Intracellular/Membrane | Not prognostic | <https://www.proteinatlas.org/ENSG00000005961-ITGA2B> |
| P16157 | ANK1_HUMAN | -6,59 | 8,82E-04 | Intracellular | Not prognostic | <https://www.proteinatlas.org/ENSG00000029534-ANK1> |
| P10720 | PF4V_HUMAN | -7,14 | 1,40E-03 | Secreted | Not prognostic | <https://www.proteinatlas.org/ENSG00000109272-PF4V1> |
| P00915 | CAH1_HUMAN | -8,47 | 1,70E-04 | Intracellular | Not prognostic | <https://www.proteinatlas.org/ENSG00000133742-CA1> |
| P02730 | B3AT_HUMAN | -12,20 | 1,70E-04 | Intracellular/Membrane | Not prognostic | <https://www.proteinatlas.org/ENSG00000004939-SLC4A1> |
