# Supplementary material for: SWATH-MS based proteomic profiling of pancreatic ductal adenocarcinoma tumours reveals the interplay between the extracellular matrix and related intracellular pathways
Source: PLoS One. 2020 Oct 13;15(10):e0240453. doi: 10.1371/journal.pone.0240453 (PMC7553299; doi:10.1371/journal.pone.0240453)
Supplement: S3 Table — (DOCX) [file pone.0240453.s007.docx]

**S3 Table**. **Top single nucleotide polymorphisms (SNVs) and associated genes**

| **Genes with SNVs (All tumour vs Normal adjacent)** | | | | **Genes with SNVs (significant differentially expressed proteins)** | | | |
| --- | --- | --- | --- | --- | --- | --- | --- |
| **Associated Gene Name** | **Chromo­some Name** | **Gene Start (bp)** | **Gene End (bp)** | **Associated Gene Name** | **Chromo­some Name** | **Gene Start (bp)** | **Gene End (bp)** |
| PIN1P1 | 1 | 70385005 | 70386000 | ESYT2 | 7 | 158523686 | 158622944 |
| NFASC | 1 | 204797779 | 204991950 | SH3PXD2B | 5 | 171752185 | 171881527 |
| PITHD1 | 1 | 24104895 | 24114722 | ISLR | 15 | 74466012 | 74469213 |
| LRRC7 | 1 | 70034081 | 70617628 | WDR1 | 4 | 10075963 | 10118573 |
| MTR | 1 | 236958610 | 237067281 | SORBS2 | 4 | 186506598 | 186877806 |
| POU2F1 | 1 | 167190066 | 167396582 | AP2A2 | 11 | 924894 | 1012239 |
| PRKG1 | 10 | 52750945 | 54058110 | CA1 | 8 | 86239837 | 86291243 |
| AC005037.3 | 2 | 201827986 | 201873825 | CA2 | 8 | 86376081 | 86393722 |
| NDUFB3 | 2 | 201936156 | 201950473 | HBD | 11 | 5253908 | 5256600 |
| AC079145.4 | 2 | 20189978 | 20203971 | SLC4A1 | 17 | 42325753 | 42345509 |
| DPP10 | 2 | 115199876 | 116603328 | CAT | 11 | 34460472 | 34493609 |
| ACOXL | 2 | 111490150 | 111875799 | TUBB | HSCHR6_MHC_APD | 30696223 | 30701448 |
| CDCA7 | 2 | 174219548 | 174233725 | TUBB | HSCHR6_MHC_QBL | 30677512 | 30682738 |
| TANC1 | 2 | 159825146 | 160089170 | TUBB | HSCHR6_MHC_DBB | 30678239 | 30683464 |
| TTC7A | 2 | 47143296 | 47303276 | TUBB | HSCHR6_MHC_MANN | 30732675 | 30737900 |
| ARHGEF4 | 2 | 131594489 | 131804836 | TUBB | HSCHR6_MHC_SSTO | 30679427 | 30684652 |
| ATG7 | 3 | 11313995 | 11599139 | TUBB | HSCHR6_MHC_MCF | 30766464 | 30771689 |
| EPHB1 | 3 | 134316643 | 134979309 | TUBB | 6 | 30687978 | 30693203 |
| GRM7 | 3 | 6811688 | 7783215 | TUBB | HSCHR6_MHC_COX | 30677842 | 30683067 |
| PRKCI | 3 | 169940153 | 170023769 | BPGM | 7 | 134331560 | 134364565 |
| NRG1 | 8 | 31496902 | 32622548 | CTSB | 8 | 11700033 | 11726957 |
| MVB12B | 9 | 129089128 | 129269320 | HMBS | 11 | 118955576 | 118964259 |
| CNTLN | 9 | 17134980 | 17503921 | HMBS | HG299_PATCH | 118955575 | 118964258 |
|  | | | | ITGA2B | 17 | 42449548 | 42466873 |
|  |  |  |  | UCHL1 | 4 | 41258430 | 41270472 |
|  |  |  |  | HIST1H1E | 6 | 26156559 | 26157343 |
|  |  |  |  | PF4V1 | 4 | 74718906 | 74719872 |
|  |  |  |  | SLC2A1 | 1 | 43391052 | 43424530 |
|  |  |  |  | ACTN1 | 14 | 69340860 | 69446157 |
|  |  |  |  | ALAD | 9 | 116148597 | 116163613 |
|  |  |  |  | LCP1 | 13 | 46700055 | 46786006 |
|  |  |  |  | PLS3 | X | 114795501 | 114885181 |
|  |  |  |  | PLS3 | HG1462_PATCH | 114797644 | 114887331 |
|  |  |  |  | ENO3 | 17 | 4851387 | 4860426 |
|  |  |  |  | ANK1 | 8 | 41510739 | 41754280 |
|  |  |  |  | ACADS | 12 | 121163538 | 121177811 |
|  |  |  |  | BPI | 20 | 36888551 | 36965907 |
|  |  |  |  | CAPN2 | 1 | 223889295 | 223963720 |
|  |  |  |  | BGN | X | 152760397 | 152775012 |
|  |  |  |  | BGN | HG1497_PATCH | 152663411 | 152678026 |
|  |  |  |  | TGM2 | 20 | 36756863 | 36794980 |
|  |  |  |  | ATP2B4 | 1 | 203595689 | 203713209 |
|  |  |  |  | BLVRB | 19 | 40953696 | 40971747 |
|  |  |  |  | SERPINB1 | 6 | 2832566 | 2842240 |
|  |  |  |  | PRDX2 | 19 | 12907634 | 12912694 |
|  |  |  |  | MYH9 | 22 | 36677327 | 36784063 |
|  |  |  |  | MYH10 | 17 | 8377523 | 8534079 |
|  |  |  |  | LIMS1 | 2 | 109150857 | 109303702 |
|  | | | | GCLC | 6 | 53362139 | 53481768 |
|  |  |  |  | CAMP | 3 | 48264837 | 48266981 |
|  |  |  |  | SERPINB8 | 18 | 61637159 | 61672278 |
|  |  |  |  | CRIP2 | 14 | 105939299 | 105946499 |
|  |  |  |  | CRIP2 | HG1592_PATCH | 105939299 | 105946499 |
|  |  |  |  | CTSC | 11 | 88026760 | 88070955 |
|  |  |  |  | BCAT1 | 12 | 24964295 | 25102393 |
|  |  |  |  | B2M | 15 | 45003675 | 45011075 |
|  |  |  |  | UFM1 | 13 | 38923986 | 38937140 |
|  |  |  |  | ACTA2 | 10 | 90694831 | 90751147 |
|  |  |  |  | GNB1 | 1 | 1716729 | 1822495 |
|  |  |  |  | GNB2 | 7 | 100271154 | 100276797 |
|  |  |  |  | RPL39 | X | 118920467 | 118925606 |
|  |  |  |  | ACTG1 | HG271_PATCH | 79483771 | 79497647 |
|  |  |  |  | ACTG1 | 17 | 79476997 | 79490873 |
|  |  |  |  | HBB | 11 | 5246694 | 5250625 |
|  |  |  |  | HBA2 | 16 | 222846 | 223709 |
|  |  |  |  | HBA1 | 16 | 226679 | 227521 |
|  |  |  |  | MPP1 | X | 154006959 | 154049282 |
|  |  |  |  | MPP1 | HG1497_PATCH | 153947152 | 153989475 |
|  |  |  |  | CAP1 | 1 | 40505905 | 40538321 |
|  |  |  |  | DMTN | 8 | 21906506 | 21940038 |
|  |  |  |  | PTK7 | 6 | 43044006 | 43129457 |
|  |  |  |  | ILK | 11 | 6624961 | 6632102 |
|  |  |  |  | TUBB2A | 6 | 3153903 | 3157760 |
|  |  |  |  | 44080 | X | 118749687 | 118827333 |
|  |  |  |  | PLS1 | 3 | 142315229 | 142432506 |
|  |  |  |  | MVP | 16 | 29831715 | 29859355 |
|  |  |  |  | LTBP1 | 2 | 33172039 | 33624576 |
|  |  |  |  | PLEC | HG104_HG975_PATCH | 144997317 | 145058904 |
|  |  |  |  | PLEC | 8 | 144989321 | 145050902 |
|  |  |  |  | DPYSL2 | 8 | 26371791 | 26515694 |
|  |  |  |  | FSCN1 | 7 | 5632439 | 5646286 |
|  |  |  |  | TUBB8 | HG905_PATCH | 96892 | 100113 |
|  |  |  |  | TUBB8 | 10 | 92828 | 120103 |
|  |  |  |  | ZC3HAV1 | 7 | 138728266 | 138794465 |
|  |  |  |  | MYPN | 10 | 69865912 | 69971774 |
|  |  |  |  | KBTBD2 | 7 | 32907784 | 32933743 |
|  |  |  |  | ALDH16A1 | 19 | 49956426 | 49974305 |
|  |  |  |  | NDUFAF2 | 5 | 60240956 | 60448853 |
|  |  |  |  | PALLD | 4 | 169418217 | 169849608 |
|  |  |  |  | EFHD2 | 1 | 15736391 | 15756839 |
|  |  |  |  | SNX18 | 5 | 53813589 | 53842415 |
|  | | | | TUBB6 | 18 | 12307668 | 12344319 |
|  |  |  |  | GBP3 | 1 | 89472349 | 89488577 |
|  |  |  |  | OLFML3 | 1 | 114522063 | 114524876 |
|  |  |  |  | LMCD1 | 3 | 8543393 | 8609805 |
|  |  |  |  | LIMA1 | 12 | 50569571 | 50677329 |
|  |  |  |  | MACF1 | 1 | 39546988 | 39952849 |
|  |  |  |  | FARP1 | 13 | 98794816 | 99102027 |
|  |  |  |  | EMILIN1 | 2 | 27301435 | 27309271 |
